# Angiotensin-Converting Enzyme 2 Protein Is Overexpressed in a Wide Range of Human Tumour Types: A Systematic Tissue Microarray Study on >15,000 Tumours

**DOI:** 10.3390/biomedicines9121831

**Published:** 2021-12-03

**Authors:** Jan Meiners, Kristina Jansen, Natalia Gorbokon, Franziska Büscheck, Andreas M. Luebke, Martina Kluth, Claudia Hube-Magg, Doris Höflmayer, Sören Weidemann, Christoph Fraune, Katharina Möller, Christian Bernreuther, Patrick Lebok, Anne Menz, Frank Jacobsen, Till Clauditz, Guido Sauter, Ria Uhlig, Waldemar Wilczak, Jakob Izbicki, Daniel Perez, Sarah Minner, Eike Burandt, Till Krech, Andreas Marx, Ronald Simon, Stefan Steurer

**Affiliations:** 1 General, Visceral and Thoracic Surgery Department and Clinic, University Medical Centre Hamburg-Eppendorf, 20246 Hamburg, Germany; j.meiners@uke.de (J.M.); k.jansen@uke.de (K.J.); izbicki@uke.de (J.I.); d.perez@uke.de (D.P.); 2 Institute of Pathology, University Medical Centre Hamburg-Eppendorf, 20246 Hamburg, Germany; n.gorbokon@uke.de (N.G.); f.buescheck@uke.de (F.B.); luebke@uke.de (A.M.L.); m.kluth@uke.de (M.K.); c.hube@uke.de (C.H.-M.); d.hoeflmayr@uke.de (D.H.); s.weidemann@uke.de (S.W.); c.fraune@uke.de (C.F.); ka.moeller@uke.de (K.M.); c.bernreuther@uke.de (C.B.); p.lebok@uke.de (P.L.); a.menz@uke.de (A.M.); f.jacobsen@uke.de (F.J.); t.clauditz@uke.de (T.C.); g.sauter@uke.de (G.S.); r.uhlig@uke.de (R.U.); w.wilczak@uke.de (W.W.); s.minner@uke.de (S.M.); e.burandt@uke.de (E.B.); t.krech@uke.de (T.K.); andreas.marx@klinikum-fuerth.de (A.M.); s.steurer@uke.de (S.S.); 3 Clinical Centre Osnabrueck, Institute of Pathology, 49074 Osnabrueck, Germany; 4 Department of Pathology, Academic Hospital Fuerth, 90766 Fuerth, Germany

**Keywords:** angiotensin-converting enzyme 2, tissue microarray, immunohistochemistry, tumour tissue

## Abstract

Angiotensin-converting enzyme 2 (ACE2) is a regulator in the renin-angiotensin system. ACE2 expression was analysed immunohistochemically in 15,306 samples from 119 tumour types and in 608 samples of 76 normal tissue types. In normal tissue, ACE2 was most abundant in testis and corpus luteum, kidney, small intestine and capillaries of selected organs. At least an occasional weak ACE2 positivity of tumour cells was seen in 83 of 119 (70%) tumour types. ACE2 tumour cell positivity was particularly frequent in papillary (94%) and clear cell (86%) renal cell carcinoma, colorectal adenocarcinoma (81%), mucinous ovarian cancer (61%), cholangiocarcinoma (58%), hepatocellular carcinoma (56%), and in adenocarcinomas of the stomach (47%), pancreas (42%), and the lung (35%). ACE2-positive capillaries were found in 409/12,644 (3%) of analysable tumours, most frequently in tumours with endocrine/neuroendocrine activity. Presence of ACE2-positive capillaries was linked to low stage in papillary thyroid cancer and low grade in neuroendocrine neoplasms. In conclusion, ACE2 expression can occur both in tumour cells and tumour-associated capillaries in a broad variety of different tumour types at highly variable frequencies.

## 1. Introduction

The angiotensin-converting enzyme 2 (ACE2) carboxypeptidase is located at the surface of endothelial cells and several other cell types [1,2,3,4,5]. The well-known function of ACE2 is to counterbalance the angiotensin-converting enzyme (ACE) by cleaving the carboxyl-terminal amino acids leucine from angiotensin I to produce angiotensin 1–9 and phenylalanine from angiotensin II to hydrolyse it into the vasodilator angiotensin 1–7 [1,2,6]. Due to its exopeptidase function, ACE2 can also cleave numerous other peptides, including des-Arg(9)-bradykinin, pyr-apelin-13 and apelin-17, neurotensin 1–8, dynorphin A 1–13, and ghrelin [1,6].

Previous analyses of normal tissues have identified ACE2 protein expression in at least one cell type of the kidney, testis, placenta, stomach, duodenum, small intestine, colon, rectum, gallbladder, liver, pancreas, thyroid gland, heart muscle, few cells in the respiratory epithelium, and endothelial cells of small vessels [5]. RNA-based analyses have also demonstrated that ACE2 expression occurs to a varying extent in miscellaneous cancer types [7,8,9,10], and upregulation of ACE2 has been linked to adverse cancer features in renal clear cell carcinoma, breast cancer, thyroid cancers and gallbladder carcinoma [11,12,13,14]. It has also been speculated that—in addition to a stressed immune system and poor general health status of cancer patients—presence of a large number of ACE2-expressing cancer cells might facilitate virus replication and contribute to the high COVID-19 mortality of cancer patients [15,16,17].

Comprehensive immunohistochemical studies on ACE2 protein expression in cancer are lacking. Therefore, we analysed our existing tissue microarrays (TMA) of cancerous samples [18] for the prevalence and significance of ACE2 protein expression.

## 2. Materials and Methods

### 2.1. Tissue Microarray

Our TMA with normal tissue has been described previously by Weidemann et al. [19]. It contained a total of 608 samples (8 samples from different donors for each of 76 different tissue types). The TMAs with cancerous samples contained a total of 15,306 primary tumours from 119 tumour types and subtypes (Supplementary Table S1). Detailed histopathological and molecular data were available for invasive breast carcinoma of no special type (*n* = 1097), clear cell renal cell carcinomas (*n* = 564), colorectal adenocarcinoma (*n* = 1554), serous ovarian carcinomas (*n* = 314), endometroid endometrial carcinomas (*n* = 168), ductal adenocarcinoma of the pancreas (*n* = 388), and thyroid carcinomas (*n* = 333). Clinical follow up data were accessible from 982 invasive breast carcinomas of no special type and 531 clear cell renal cell cancer patients with a median follow-up time of 50/40 months (range 1–88/1–250). All samples were from the archives of the Institutes of Pathology, University Hospital of Hamburg, Germany, the Institute of Pathology, Clinical Centre Osnabrueck, Germany, and Department of Pathology, Academic Hospital Fuerth, Germany. Tissues were fixed in 4% buffered formalin and then embedded in paraffin. The TMA manufacturing process has previously been described in detail [20,21,22]. In brief, one tissue spot (diameter: 0.6 mm) was transmitted from a representative cancer-containing donor block in an empty recipient paraffin block. The use of archived remnants of diagnostic tissues for TMA manufacturing, their analysis for research purposes, and patient data were according to local laws (HmbKHG, §12).

### 2.2. Immunohistochemistry

TMAs were immunostained as described in Menz et al. [22]. Primary antibody specific for ACE2 (recombinant rabbit, MSVA-919R MS Validated Antibodies, GmbH, Hamburg, Germany) was applied at 37 °C for 60 min at a dilution of 1:60. Bound antibody was then visualised using the EnVision Kit™ (Agilent, Santa Clara, CA, USA; #K5007) according to the manufacturer’s directions. Normal human lymphatic tissue (lymph node) was used as a negative control. For tumour tissues, the percentage of positive neoplastic cells was estimated, and the staining intensi-+ty was semi-quantitatively recorded (0, 1+, 2+, 3+). For statistical analyses, the staining results were categorised into four groups. Tumours without any staining were considered negative. Tumours with 1+ staining intensity in ≤70% of cells and 2+ intensity in ≤30% of cells were considered weakly positive. Tumours with 1+ staining intensity in >70% of cells, 2+ intensity in 31–70%, or 3+ intensity in ≤30% were considered moderately positive. Tumours with 2+ intensity in >70% or 3+ intensity in >30% of cells were considered strongly positive. Presence or absence of ACE2 stained vessels was also recorded as “negative”, “few”, and “many”.

### 2.3. Statistics

Contingency tables and chi^2^-tests were performed to search for associations between ACE2 and tumour phenotype. Survival curves were calculated according to Kaplan–Meier. The Log-Rank test was applied to detect significant differences between groups. JMP 14 was used (SAS Institute Inc., Cary, NC, USA). A *p*-value of ≤0.05 was considered statistically significant.

## 3. Results

### 3.1. ACE2 in Normal Tissue

**Figure 1:**
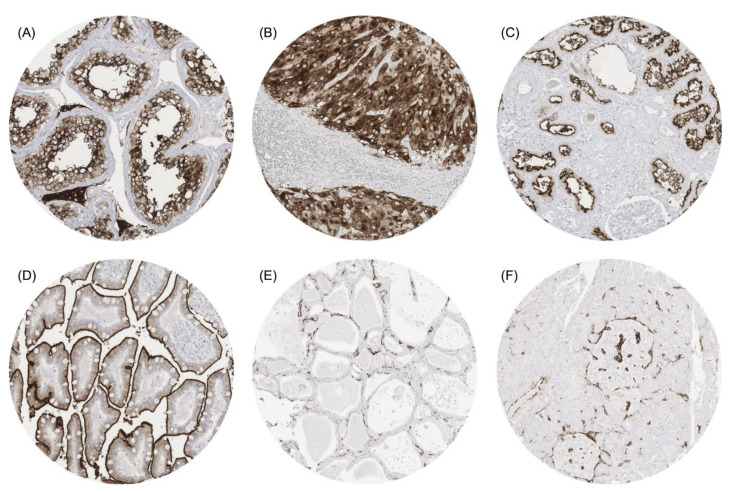
ACE2 staining of normal tissues. The panels show strong ACE2 positivity of Leydig cells and spermatocytes in the testis (**A**), corpus luteum of the ovary (**B**), proximal tubuli of the kidney (**C**), and surface epithelium of the small intestine mucosa (**D**). ACE2 vessel staining is seen in the thyroid gland (**E**) and in the pancreas where the staining is particularly strong in the surroundings of islets of Langerhans (**F**). Magnification 100×, TMA spot size 600 µm.

The strongest ACE2 immunostaining was seen in Leydig cells and spermatocytes in the testis (Figure 1A), corpus luteum of the ovary (Figure 1B), proximal tubuli of the kidney (Figure 1C), surface epithelium of the small intestine (Figure 1D), and of the gallbladder. A distinct but clearly weaker ACE2 positivity was observed in a fraction of heart muscle fibres, sebaceous glands, syncytiotrophoblast, cytotrophoblast and chorion cells of the placenta, decidua cells, adrenal cortical cells, superficial cell layer of the anal canal transitional epithelium, the parietal layer of the Bowman capsule of the kidney, glands of the stomach antrum, and the surface epithelium of the colon. Few cells of showed weak to moderate staining of apical cell membrane in alveolar pneumocytes, respiratory epithelium of the bronchus and nasal sinuses, fallopian tube, epididymis, and seminal vesicle (strong positivity in few cells). In addition, endothelial cells of small blood vessels showed a variable ACE2 positivity in an organ-specific manner with highest expression levels in endocrine organs (Figure 1E) and the heart. In the pancreas, ACE2 vessel staining was stronger in and around islets of Langerhans, than in the exocrine pancreas (Figure 1F). ACE2 immunostaining was not visible in various other organs, for example in the brain, pituitary gland, squamous epithelium, urothelium, lymphatic tissues, bone marrow, hepatocytes, epithelial cells of the pancreas, salivary glands, Brunner glands, bronchial glands, breast, endometrium, endocervix, smooth muscle, and skeletal or smooth muscle.

### 3.2. ACE2 in Cancer

**Figure 2:**
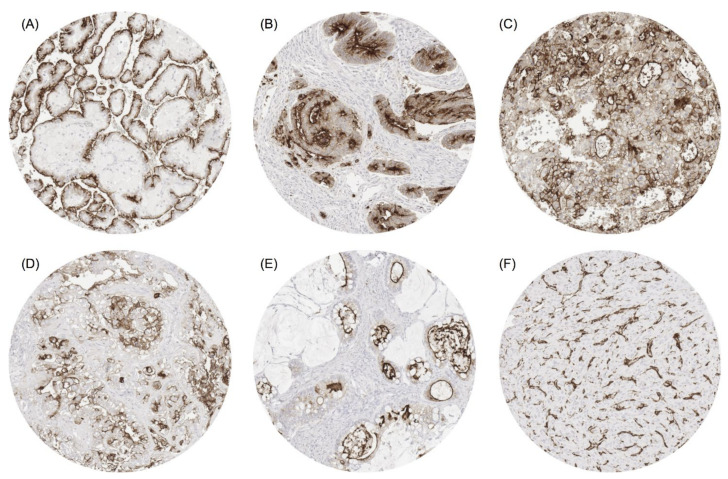
ACE2 staining in cancer. The panels show strong or moderate ACE2 positivity in cases of papillary renal cell carcinoma (**A**), colorectal adenocarcinoma (**B**), hepatocellular carcinoma (**C**), clear cell carcinoma (**D**) and mucinous carcinoma of the ovary (**E**). Panel (**F**) shows an ACE2-negative medullary carcinoma of the thyroid containing ACE2-positive capillaries. Magnification 100×, TMA spot size 600 µm.

A positive ACE2 immunostaining was detectable in 3542 (28.0%) of the 12,644 analysable tumours, including 2029 (16.0%) with weak, 708 (5.6%) with moderate and 805 (6.4%) with strong immunostaining. Overall, 83 (69.7%) of 119 tumour categories showed a detectable ACE2 expression, with 53 (44.5%) tumour categories showing at least in one case a moderate positivity and 37 (31.1%) tumour categories with at least one strongly positive case (Supplementary Table S1). Representative images of ACE2-positive tumours are shown in Figure 2.

**Figure 3:**
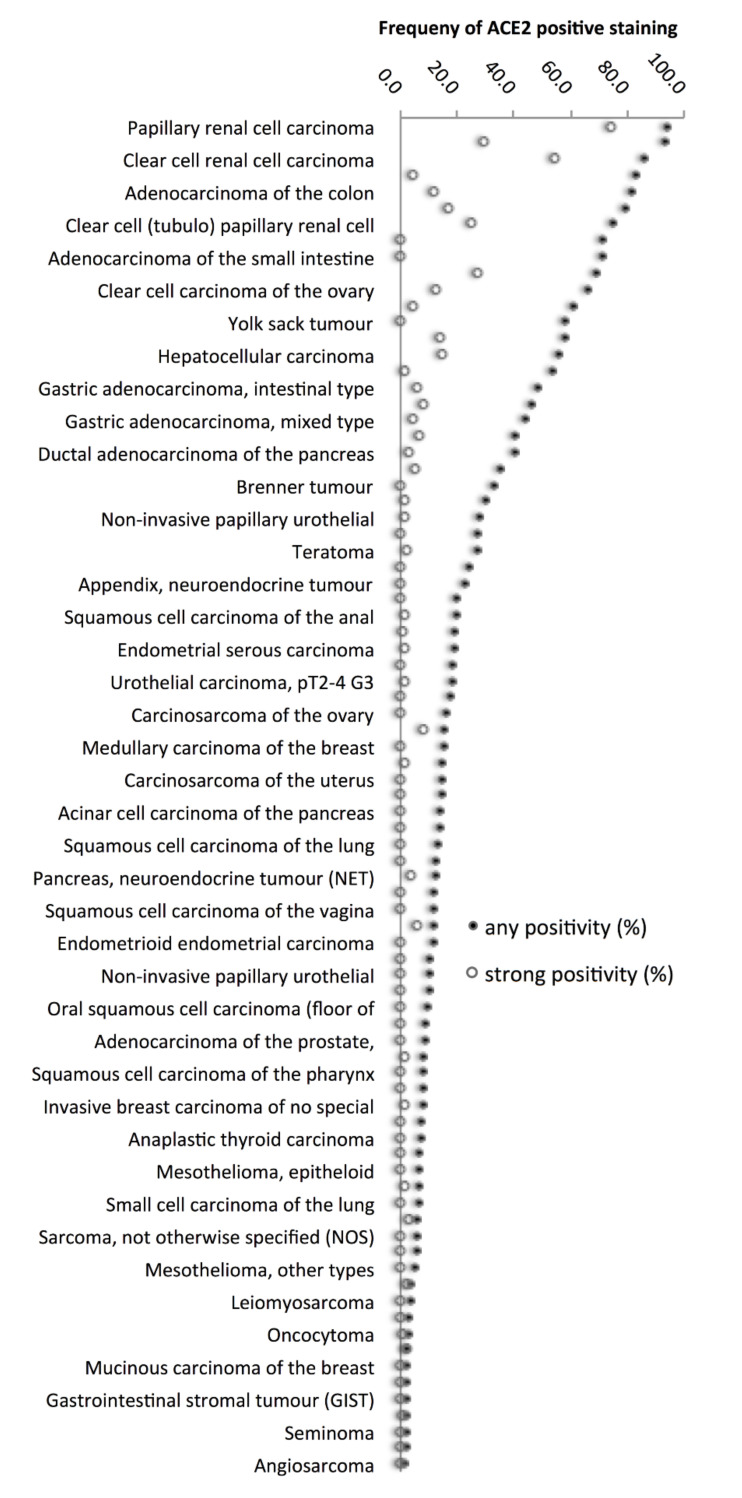
Ranking order of ACE2 staining in human tumours. Both the frequency of positive cases (filled circles) and the frequency of strongly positive cases (open circles) are shown. Thirty-nine additional tumour entities without any ACE2-positive cases are not shown.

The highest rate of positive staining was found in kidney tumours arising from the proximal tubulus (papillary (94.0%), and clear cell (85.8%)), followed by colorectal adenocarcinomas (81.3%), clear cell carcinomas from other origins than the kidney (65.9–71.4%), cholangiocellular (57.9%) and hepatocellular carcinomas (56.1%) of the liver, as well as adenocarcinomas of the stomach (43.8–48.6%) and the pancreas (40.3–53.9%). A graphical representation of a ranking order of ACE2-positive and strongly positive cancers is given in Figure 3.

ACE2 positivity in tumour-associated blood vessels was found in 409 (3.2%) of the 12,644 analysable tumours, including 283 (69.2%) with few and 126 (30.8%) with many recorded vessels. A total of 33 (27.0%) of 119 tumour categories showed an ACE2 positivity of small vessels in at least one case. The highest rates of vessel positivity occurred in tumours with endocrine or neuroendocrine activity such as thyroid cancers and neuroendocrine tumours (Table 1).

### 3.3. ACE2, Cancer Phenotype and Prognosis

The comparison of ACE2 expression, histopathological and molecular data in colorectal, kidney, breast, pancreas, ovarian, endometrial, and thyroid cancer revealed that the impact of ACE2 expression on cancer aggressiveness varied between tumour entities (Table 2).

**Figure 4:**
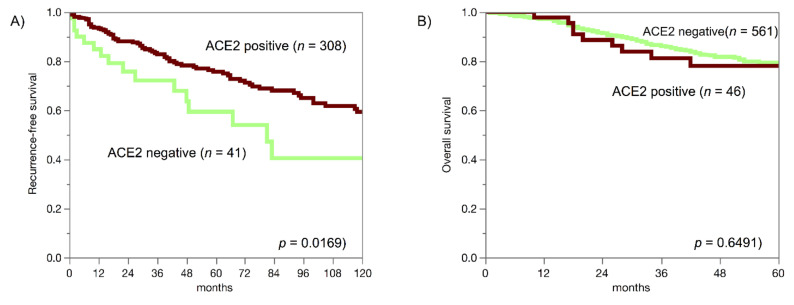
ACE2 staining and prognosis in clear cell renal cell carcinomas (**A**) and invasive breast carcinoma of no special type (**B**).

Reduced ACE2 expression was linked to unfavourable tumour phenotype in colorectal adenocarcinoma and in clear cell renal cell carcinoma and linked to reduced recurrence-free survival in clear cell renal cell carcinoma (*p* = 0.0169; Figure 4A). In invasive breast carcinoma of no special type, elevated ACE2 immunostaining was linked to high grade, HER2 overexpression and loss of oestrogen and progesterone receptors (*p* < 0.0001 each) but was unrelated to patient outcome in the largest subgroup of invasive breast carcinomas of no special type (*p* = 0.6491; Figure 4B).

ACE2 immunostaining in tumour cells was unrelated to tumour phenotype in ovarian, endometrial and thyroid carcinomas. Presence of ACE2-positive small blood vessels was linked to low pathological tumour stage (pT) in papillary thyroid carcinomas (*p* = 0.0446) and more commonly seen in neuroendocrine tumours than in neuroendocrine carcinomas (*p* = 0.0006; Table 3).

## 4. Discussion

Our extensive normal tissue analysis involving 76 different tissue categories identified testes, corpus luteum of the ovary, kidney and the small intestine as tissues with the highest ACE2 expression levels. These observations and most other normal tissue findings fit well with the results of a previous comprehensive study by Hikmet et al. analysing 46 normal tissue types by two different antibodies and comparing the data with RNA expression levels [5]. The only differences between this and our study included ACE2 positivity in adrenal gland and corpus luteum, which was not seen (adrenal gland) or not analysed (corpus luteum) by Hikmet et al. Adrenal gland staining was considered a potential cross-reactivity, because RNA expression has not been described for this organ [4,5], and analyses of adrenal gland tumours were therefore not performed in this study.

The particularly high ACE2 expression in critical organs for fertility in combination with the prominent role of ACE2 as an entry point for the coronavirus causing COVID-19 fits well to reports on tissue damage [4] and decreased sperm concentration and motility in many patients who recovered from severe or moderate courses of COVID-19 (summarised in [23]). The particularly high expression of ACE2 in corpora lutea of the ovary, which have a key function for preserving pregnancy during the first trimester, is further in line with reports describing individual cases of miscarriage during this critical time span in COVID-19 patients [24].

Our analysis of 12,664 tumours of 119 different subtypes identified renal cell carcinomas derived from the proximal tubulus and colorectal adenocarcinomas as the most frequently ACE2-positive cancer types, followed by gastric adenocarcinoma, ductal and papilla Vaterii-derived pancreatic adenocarcinoma as well as cholangiocellular and hepatocellular carcinoma of the liver. This finding is in complete agreement with data from “The Cancer Genome Atlas” (TCGA) summarised in two recent publications [7,10]. Other tumour entities which were often ACE2-positive were too rare for being separately analysed within ICGC/TCGA studies, for example clear cell carcinomas of the ovary and the endometrium which resemble the commonly positive clear cell carcinomas of the kidney and mucinous carcinomas of the ovary which resemble the commonly positive colorectal adenocarcinomas. That a moderate to strong ACE2 immunostaining was found in 53 of our 119 tumour categories (45%) may suggest that this enzyme can exert a biologically relevant role in many different neoplastic cell types. Of note, the exceptional low rate of ACE2 positivity in chromophobe renal cell cancers (4%) as compared to clear cell (86%) or papillary (94%) renal cell cancers suggests that ACE2 immunohistochemistry may be helpful for renal cell cancer subtype diagnosis.

The availability of data on histopathologic parameters of tumour aggressiveness or clinical follow-up data for several of our tumour cohorts enabled us to further investigate a potential biological or clinical effect of ACE2 expression in cancer cells. Our observation of a tumour type-dependent relationship between high or low ACE2 expression levels and tumour aggressiveness is concordant with data from previous studies interrogating existing RNA expression databases [7,8,9]. Based on the TCGA database, links between high ACE2 expression and a favourable prognosis in clear cell renal cell carcinomas [7], and in colorectal carcinomas [8] have been described. Again, concordant with our findings, Nair et al. [25] described a tendency towards poor patient prognosis in cancers with high ACE2 expression levels in an analysis of RNA expression data from a set of 2730 breast cancer patients, enriched for HER2 positive cases. Suggested mechanisms, by which elevated or decreased ACE2 protein levels could facilitate cancer progression, include stimulation of tumour angiogenesis [26] and inhibition of immune cell infiltration [27]. Alternatively, it cannot be excluded, that a loss of ACE2 in cancers derived from a normal progenitor cell that physiologically expresses ACE2—such as kidney and colon epithelium—may reflect cancer cell dedifferentiation and thus be related to unfavourable tumour behaviour even if the diminished ACE2 expression does not contribute to the altered biologic behaviour of cells. Similarly, up regulation of ACE2 in tissues that normally do not show detectable levels of ACE2 protein may constitute a phenomenon of cancer progression without an own contribution of ACE2 to increased tumour aggressiveness.

It is a distinct advantage of immunohistochemical studies over RNA-based analyses, that individual cell types can be separately analysed. For ACE2 it was conspicuous, both in normal and in neoplastic tissues that some samples showed a strong staining of small capillaries, while most other specimen did not. Since our findings in tumours paralleled the results in normal tissues and mainly involved cancers with endocrine or neuroendocrine features it appears that endothelial ACE2 expression represents a relevant property of tissues secreting active hormones into the blood. One might speculate that ACE2-induced local vasodilatation could increase the blood circulation of these tissues and by doing so facilitate the release of active substances into the blood. That the content of ACE2-positive capillaries decreased with pT stage in papillary thyroid carcinomas and was lower in neuroendocrine carcinomas than in neuroendocrine tumours would be consistent with this assumption. It appears very likely that the production of active substances secreted to the blood stream will decrease in these tumours during cancer progression. Whether or not the predilection of ACE2 expression in capillaries of endocrine organs is related to the dysfunction of these organs occurring either during COVID-19 or as a long-term effect of this disease [28,29] remains to be clarified.

## 5. Conclusions

In summary, our highly standardised analysis of more than 15,000 cancers revealed that ACE2 expression can occur both in tumour cells and tumour-associated capillaries in a broad variety of different tumour types at highly variable frequencies. Despite some associations between ACE2 expression and tumour phenotype or patient outcome that were seen in some tumour entities, the clinical impact of ACE2 expression on the clinical course of these cancers appears to be limited in most entities. Whether or not the ACE2 expression level of tumours impacts the clinical course of COVID-19-infected cancer patient’s remains to be seen.

## Figures and Tables

**Table 1:** ACE2 immunostaining in cancer-associated blood vessel.

Tumour Type	Fraction of Tumours (%) with ACE2-Positive Blood Vessels
Pancreas, neuroendocrine tumour	70.3
Adenoma of the thyroid gland	52.8
Medullary thyroid carcinoma	48.4
Ileum, neuroendocrine tumour	43.2
Lung, neuroendocrine tumour	35.3
Adrenal cortical adenoma	34.1
Follicular thyroid carcinoma	27.7
Ganglioneuroma	25.0
Pancreas, neuroendocrine carcinoma	21.4
Colorectal, neuroendocrine tumour	20.0
Colorectal, neuroendocrine carcinoma	20.0
Papillary thyroid carcinoma	16.5
Gastrointestinal stromal tumour	16.3
Oncocytoma	15.5
Chromophobe renal cell carcinoma	11.3
Paraganglioma	10.8
Leiomyoma	8.3
Adrenal cortical carcinoma	7.7
Teratoma	6.8
Embryonal carcinoma of the testis	5.0
Warthin tumour of the parotid gland	4.5
Mesothelioma, epitheloid	3.3
Pancreatic/Ampullary adenocarcinoma	2.6
Yolk sack tumour	2.6
Leiomyosarcoma	2.4
Seminoma	2.4
Adenocarcinoma of the prostate (recurrence)	2.4
Endometrioid carcinoma of the ovary	2.1
Ductal adenocarcinoma of the pancreas	1.8
Mucinous carcinoma of the ovary	1.3
Adenocarcinoma of the prostate, Gleason 5+5	1.3
Hodgkin Lymphoma	1.2
Clear cell renal cell carcinoma	0.2

**Table 2:** Cancer phenotype and ACE2 immunostaining (%).

Cancer	Phenotype	Class	N	Negative	Weak	Moderate	Strong	*p*
Invasive breast carcinoma of no special type		Total	1213	92.2	4.1	2	1.7	
	Tumour stage	pT1	599	93.8	2.7	2	1.5	0.0903
		pT2	412	90.8	5.8	1.9	1.5	
		pT3-4	86	87.2	8.1	1.2	3.5	
	Grade	G1	179	98.9	0.6	0.6	0	<0.0001
		G2	584	95.7	1.9	1	1.4	
		G3	376	83.2	9.8	3.7	3.2	
	Nodal stage	pN0	514	91.6	3.3	3.3	1.8	0.0959
		pN1	227	93.4	4.4	0.9	1.3	
		pN2	70	90	5.7	1.4	2.9	
		pN3	54	85.2	11.1	0	3.7	
	HER2 status	Negative	850	93.6	3.2	1.6	1.5	<0.0001
		Positive	119	79.8	13.4	4.2	2.5	
	ER status	Negative	202	67.8	15.8	8.4	7.9	<0.0001
		Positive	715	98.5	1.3	0.1	0.1	
	PR status	Negative	391	80.6	10.2	4.9	4.3	<0.0001
		Positive	569	99.1	0.7	0.2	0	
	Triple negative	No	753	96	2.8	0.7	0.5	<0.0001
		Yes	135	67.4	14.1	9.6	8.9	
Colorectal adenocarcinoma		Total	1645	18.7	43.5	26.2	11.7	
	Tumour stage	pT1	63	7.9	46	28.6	17.5	0.0004
		pT2	309	17.5	40.8	28.5	13.3	
		pT3	867	16.8	44.1	27.2	11.9	
		pT4	315	27.9	42.9	20.3	8.9	
	Nodal stage	pN0	798	17.9	41.6	27.8	12.7	0.1796
		pN+	741	20.2	44.7	24.3	10.8	
	Localization	Left colon	1129	16.6	42.5	26.9	14	<0.0001
		Right colon	432	25.2	45.6	23.1	6	
	MMR status	Defective	73	49.3	38.4	11	1.4	<0.0001
		Proficient	1065	14.2	44.2	28.8	12.8	
	RAS status	Mutated	302	20.9	47.7	23.2	8.3	0.0004
		Wild type	419	13.4	41.5	29.4	15.8	
	BRAF status	Mutated	18	66.7	27.8	5.6	0	0.0002
		Wild type	112	17.9	45.5	24.1	12.5	
Clear cell renal cell carcinomas		Total	572	14.3	17.8	13.6	54.2	
	ISUP stage	1	181	9.9	26.5	13.8	49.7	<0.0001
		2	182	8.8	15.4	15.4	60.4	
		3	165	18.2	9.1	13.9	58.8	
		4	36	50	27.8	2.8	19.4	
	Fuhrman grade	1	26	11.5	19.2	15.4	53.8	<0.0001
		2	332	8.4	20.2	15.1	56.3	
		3	169	17.2	13	13	56.8	
		4	44	50	18.2	4.5	27.3	
	Thoenes grade	1	202	8.9	23.8	15.3	52	<0.0001
		2	314	12.7	14.3	14	58.9	
		3	55	43.6	16.4	5.5	34.5	
	UICC stage	1	255	11.8	18.8	15.7	53.7	0.2557
		2	27	14.8	11.1	7.4	66.7	
		3	73	19.2	12.3	12.3	56.2	
		4	61	23	18	8.2	50.8	
	pT stage	pT1	333	11.4	19.2	16.5	52.9	0.068
		pT2	58	12.1	19	8.6	60.3	
		pT3-4	176	19.3	14.8	10.2	55.7	
	pN stage	pN0	98	22.4	14.3	12.2	51	0.342
		pN ≥ 1	11	27.3	0	18.2	54.5	
Endometroid endometrial carcinoma	Total		222	88.3	11.7	0	0	
	Tumour stage	pT1	111	90.1	9.9	0	0	0.3277
		pT2	24	79.2	20.8	0	0	
		pT3-4	33	84.8	15.2	0	0	
	Nodal stage	pN0	50	78	22	0	0	0.0651
		pN+	29	93.1	6.9	0	0	
Serous ovarian carcinomas	Total		474	89.7	10.3	0	0	
	Tumour stage	pT1	29	93.1	6.9	0	0	0.5892
		pT2	41	87.8	12.2	0	0	
		pT3	244	86.9	13.1	0	0	
	Nodal stage	pN0	74	90.5	9.5	0	0	0.737
		pN1	156	89.1	10.9	0	0	
Pancreatic ductal adenocarcinomas	Total		454	59.7	32.4	4.8	3.1	
	Tumour stage	pT1	13	46.2	46.2	0	7.7	0.5988
		pT2	54	57.4	31.5	5.6	5.6	
		pT3	302	63.9	29.8	4	2.3	
		pT4	19	57.9	36.8	0	5.3	
	Grade	1	13	53.8	23.1	15.4	7.7	0.612
		2	270	63	31.5	3	2.6	
		3	83	61.4	31.3	3.6	3.6	
	Nodal stage	pN0	81	69.1	25.9	2.5	2.5	0.4772
		pN+	305	60	32.5	4.3	3.3	
Thyroid carcinomas	Papillary	Total	357	92.2	7.6	0.3	0	
	Tumour stage	pT1	114	92.1	7.9	0	0	0.1363
		pT2	64	85.9	14.1	0	0	
		pT3-4	84	95.2	4.8	0	0	
	Nodal stage	pN0	76	92.1	7.9	0	0	0.126
		pN+	105	97.1	2.9	0	0	
	Follicular	Total	141	80.1	19.9	0	0	
	Tumour stage	pT1	11	72.7	27.3	0	0	0.5715
		pT2	29	69	31	0	0	
		pT3-4	31	80.6	19.4	0	0	
	Nodal stage	pN0	22	90.9	9.1	0	0	0.5462
		pN+	2	100	0	0	0	

**Table 3:** Cancer phenotype and ACE2 immunostaining in tumour-associated blood vessels (%).

Cancer	Phenotype	Class	N	Negative	Positive	*p*
Papillary thyroid carcinoma	Tumour stage	pT1	139	79.9	20.1	0.0446
		pT2	76	81.6	18.4	
		pT3-4	92	91.3	8.7	
	Nodal stage	pN0	84	89.3	10.7	0.933
		pN+	116	89.7	10.3	
Neuroendocrine tumour		Total	133	53.4	46.6	0.0006
Neuroendocrine cancer		Total	25	88	12	

## Data Availability

All data generated or analysed during this study are included in this published article and its supplementary information file.

## Supplementary Materials

The following are available online at https://www.mdpi.com/article/10.3390/biomedicines9121831/s1, Table S1: ACE2 immunostaining in human tumours.
