# Supplementary material for: Angiotensin-Converting Enzyme 2 Protein Is Overexpressed in a Wide Range of Human Tumour Types: A Systematic Tissue Microarray Study on >15,000 Tumours
Source: Biomedicines. 2021 Dec 3;9(12):1831. doi: 10.3390/biomedicines9121831 (PMC8698714; doi:10.3390/biomedicines9121831)
Supplement: Supplementary file 1 [file biomedicines-09-01831-s001.zip › biomedicines-1413108-supplementary.pdf]

Table S1: ACE2 immunostaining in human tumours.

Tumour entity	On TMA (n)	ACE2 immunostaining						
			In tumour cells				In blood vessels	
		Analysable (n)	Negative (%)	Weak (%)	Moderate (%)	Strong (%)	Negative (%)	Positive (%)
Pilomatrixoma	35	34	82.4	14.7	2.9	0.0	100.0	0.0
Basal cell carcinoma	88	52	98.1	1.9	0.0	0.0	100.0	0.0
Benign nevus	29	26	100.0	0.0	0.0	0.0	100.0	0.0
Squamous cell carcinoma of the skin	90	83	92.8	7.2	0.0	0.0	100.0	0.0
Malignant melanoma	48	43	97.7	0.0	0.0	2.3	100.0	0.0
Merkel cell carcinoma	46	43	100.0	0.0	0.0	0.0	100.0	0.0
Squamous cell carcinoma of the larynx	110	92	88.0	10.9	1.1	0.0	100.0	0.0
Squamous cell carcinoma of the pharynx	60	50	92.0	8.0	0.0	0.0	100.0	0.0
Oral squamous cell carcinoma (floor of the mouth)	130	109	90.8	9.2	0.0	0.0	100.0	0.0
Pleomorphic adenoma of the parotid gland	50	46	100.0	0.0	0.0	0.0	100.0	0.0
Warthin tumour of the parotid gland	49	44	81.8	18.2	0.0	0.0	95.5	4.5
Basal cell adenoma of the salivary gland	15	13	100.0	0.0	0.0	0.0	100.0	0.0
Adenocarcinoma of the lung	246	167	64.7	25.7	4.8	4.8	100.0	0.0
Squamous cell carcinoma of the lung	130	68	86.8	11.8	1.5	0.0	100.0	0.0
Small cell carcinoma of the lung	20	16	93.8	6.3	0.0	0.0	100.0	0.0
Mesothelioma, epitheloid	39	30	93.3	6.7	0.0	0.0	96.7	3.3
Mesothelioma, other types	76	63	95.2	4.8	0.0	0.0	100.0	0.0
Thymoma	29	28	100.0	0.0	0.0	0.0	100.0	0.0
Squamous cell carcinoma of the vagina	78	67	88.1	11.9	0.0	0.0	100.0	0.0
Squamous cell carcinoma of the vulva	130	115	85.2	13.0	0.0	1.7	100.0	0.0

Squamous cell carcinoma of the cervix	130	124	75.8	22.6	1.6	0.0	100.0	0.0
Endometrioid endometrial carcinoma	236	222	88.3	11.7	0.0	0.0	100.0	0.0
Endometrial serous carcinoma	82	73	80.8	13.7	4.1	1.4	100.0	0.0
Carcinosarcoma of the uterus	48	41	85.4	14.6	0.0	0.0	100.0	0.0
Endometrial carcinoma, high grade, G3	13	13	84.6	7.7	0.0	7.7	100.0	0.0
Endometrial clear cell carcinoma	8	7	28.6	42.9	28.6	0.0	100.0	0.0
Endometrioid carcinoma of the ovary	110	94	70.2	28.7	0.0	1.1	97.9	2.1
Serous carcinoma of the ovary	559	474	89.7	10.3	0.0	0.0	100.0	0.0
Mucinous carcinoma of the ovary	96	75	38.7	49.3	8.0	4.0	98.7	1.3
Clear cell carcinoma of the ovary	50	41	34.1	39.0	14.6	12.2	100.0	0.0
Carcinosarcoma of the ovary	47	38	84.2	15.8	0.0	0.0	100.0	0.0
Brenner tumour	9	9	66.7	33.3	0.0	0.0	100.0	0.0
Invasive breast carcinoma of no special type	1391	1213	92.2	4.1	2.0	1.7	100.0	0.0
Lobular carcinoma of the breast	294	246	98.0	0.8	0.4	0.8	100.0	0.0
Medullary carcinoma of the breast	26	26	84.6	11.5	3.8	0.0	100.0	0.0
Tubular carcinoma of the breast	27	24	100.0	0.0	0.0	0.0	100.0	0.0
Mucinous carcinoma of the breast	58	43	97.7	2.3	0.0	0.0	100.0	0.0
Phyllodes tumour of the breast	50	50	100.0	0.0	0.0	0.0	100.0	0.0
Adenomatous polyp, low-grade dysplasia	50	47	17.0	72.3	6.4	4.3	100.0	0.0
Adenomatous polyp, high-grade dysplasia	50	48	20.8	45.8	16.7	16.7	100.0	0.0
Adenocarcinoma of the colon	1932	1645	18.7	43.5	26.2	11.7	100.0	0.0
Gastric adenocarcinoma, diffuse type	226	141	53.9	33.3	5.0	7.8	100.0	0.0
Gastric adenocarcinoma, intestinal type	224	144	51.4	34.0	9.0	5.6	100.0	0.0
Gastric adenocarcinoma, mixed type	62	48	56.3	33.3	6.3	4.2	100.0	0.0
Adenocarcinoma of the oesophagus	133	64	59.4	31.3	3.1	6.3	100.0	0.0
Squamous cell carcinoma of the oesophagus	124	44	93.2	6.8	0.0	0.0	100.0	0.0
Squamous cell carcinoma of the anal canal	91	77	80.5	15.6	2.6	1.3	100.0	0.0
Cholangiocarcinoma	114	95	42.1	34.7	9.5	13.7	100.0	0.0

Hepatocellular carcinoma	50	41	43.9	31.7	9.8	14.6	100.0	0.0
Ductal adenocarcinoma of the pancreas	662	454	59.7	32.4	4.8	3.1	98.2	1.8
Pancreatic/Ampullary adenocarcinoma	119	76	46.1	40.8	11.8	1.3	97.4	2.6
Acinar cell carcinoma of the pancreas	15	14	85.7	14.3	0.0	0.0	100.0	0.0
Gastrointestinal stromal tumour (GIST)	50	49	98.0	0.0	2.0	0.0	83.7	16.3
Non-invasive papillary urothelial carcinoma, pTa G2 low grade	177	146	89.7	9.6	0.7	0.0	100.0	0.0
Non-invasive papillary urothelial carcinoma, pTa G2 high grade	141	124	85.5	13.7	0.8	0.0	100.0	0.0
Non-invasive papillary urothelial carcinoma, pTa G3	187	164	72.0	24.4	2.4	1.2	100.0	0.0
Urothelial carcinoma, pT2-4 G3	624	532	82.0	15.0	1.5	1.5	100.0	0.0
Small cell neuroendocrine carcinoma of the bladder	18	18	94.4	5.6	0.0	0.0	100.0	0.0
Sarcomatoid urothelial carcinoma	25	24	87.5	12.5	0.0	0.0	100.0	0.0
Clear cell renal cell carcinoma	858	620	14.2	17.7	14.0	54.0	99.8	0.2
Papillary renal cell carcinoma	255	183	6.0	10.4	9.3	74.3	100.0	0.0
Clear cell (tubulo) papillary renal cell carcinoma	21	16	25.0	25.0	25.0	25.0	100.0	0.0
Chromophobe renal cell carcinoma	131	106	96.2	1.9	0.0	1.9	88.7	11.3
Oncocytoma	177	129	96.9	1.6	0.8	0.8	84.5	15.5
Adenocarcinoma of the prostate, Gleason 3+3	83	80	91.3	7.5	1.3	0.0	100.0	0.0
Adenocarcinoma of the prostate, Gleason 4+4	80	73	91.8	6.8	0.0	1.4	100.0	0.0
Adenocarcinoma of the prostate, Gleason 5+5	85	78	93.6	5.1	0.0	1.3	98.7	1.3
Adenocarcinoma of the prostate (recurrence)	261	212	80.7	17.9	0.9	0.5	97.6	2.4
Small cell neuroendocrine carcinoma of the prostate	17	16	100.0	0.0	0.0	0.0	100.0	0.0
Seminoma	621	546	98.0	1.8	0.0	0.2	97.6	2.4
Embryonal carcinoma of the testis	50	40	72.5	27.5	0.0	0.0	95.0	5.0
Yolk sack tumour	50	38	42.1	57.9	0.0	0.0	97.4	2.6
Teratoma	50	44	72.7	18.2	6.8	2.3	93.2	6.8
Squamous cell carcinoma of the penis	80	66	90.9	7.6	1.5	0.0	100.0	0.0
Adenoma of the thyroid gland	114	106	85.8	13.2	0.9	0.0	47.2	52.8
Papillary thyroid carcinoma	392	357	92.2	7.6	0.3	0.0	83.5	16.5

Follicular thyroid carcinoma	158	141	80.1	19.9	0.0	0.0	72.3	27.7
Medullary thyroid carcinoma	107	95	96.8	3.2	0.0	0.0	51.6	48.4
Anaplastic thyroid carcinoma	45	42	92.9	7.1	0.0	0.0	100.0	0.0
Phaeochromocytoma	50	44	100.0	0.0	0.0	0.0	100.0	0.0
Appendix, neuroendocrine tumour (NET)	22	13	76.9	15.4	7.7	0.0	100.0	0.0
Colorectal, neuroendocrine tumour (NET)	11	10	90.0	10.0	0.0	0.0	80.0	20.0
Ileum, neuroendocrine tumour (NET)	49	44	97.7	2.3	0.0	0.0	56.8	43.2
Lung, neuroendocrine tumour (NET)	19	17	88.2	5.9	0.0	5.9	64.7	35.3
Pancreas, neuroendocrine tumour (NET)	98	91	87.9	5.5	3.3	3.3	29.7	70.3
Colorectal, neuroendocrine carcinoma (NEC)	12	10	100.0	0.0	0.0	0.0	80.0	20.0
Gallbladder, neuroendocrine carcinoma (NEC)	4	4	100.0	0.0	0.0	0.0	100.0	0.0
Pancreas, neuroendocrine carcinoma (NEC)	14	14	100.0	0.0	0.0	0.0	78.6	21.4
Hodgkin Lymphoma	103	81	100.0	0.0	0.0	0.0	98.8	1.2
Non-Hodgkin Lymphoma	62	52	100.0	0.0	0.0	0.0	100.0	0.0
Small lymphocytic lymphoma, B-cell type (B-SLL/B-CLL)	50	32	100.0	0.0	0.0	0.0	100.0	0.0
Diffuse large B cell lymphoma (DLBCL)	114	98	100.0	0.0	0.0	0.0	100.0	0.0
Follicular lymphoma	88	66	100.0	0.0	0.0	0.0	100.0	0.0
T-cell Non Hodgkin lymphoma	24	18	100.0	0.0	0.0	0.0	100.0	0.0
Mantle cell lymphoma	18	14	100.0	0.0	0.0	0.0	100.0	0.0
Marginal zone lymphoma	16	11	100.0	0.0	0.0	0.0	100.0	0.0
Diffuse large B-cell lymphoma (DLBCL) in the testis	16	14	100.0	0.0	0.0	0.0	100.0	0.0
Burkitt lymphoma	5	1	100.0	0.0	0.0	0.0	100.0	0.0
Tenosynovial giant cell tumour	45	44	100.0	0.0	0.0	0.0	100.0	0.0
Granular cell tumour	53	46	100.0	0.0	0.0	0.0	100.0	0.0
Leiomyoma	50	48	100.0	0.0	0.0	0.0	91.7	8.3
Leiomyosarcoma	87	82	96.3	2.4	1.2	0.0	97.6	2.4
Liposarcoma	132	127	100.0	0.0	0.0	0.0	100.0	0.0
Malignant peripheral nerve sheath tumour (MPNST)	13	11	100.0	0.0	0.0	0.0	100.0	0.0

Myofibrosarcoma	26	26	100.0	0.0	0.0	0.0	100.0	0.0
Angiosarcoma	73	63	98.4	1.6	0.0	0.0	100.0	0.0
Angiomyolipoma	91	91	100.0	0.0	0.0	0.0	100.0	0.0
Dermatofibrosarcoma protuberans	21	18	100.0	0.0	0.0	0.0	100.0	0.0
Ganglioneuroma	14	12	100.0	0.0	0.0	0.0	75.0	25.0
Kaposi sarcoma	8	6	100.0	0.0	0.0	0.0	100.0	0.0
Neurofibroma	117	88	100.0	0.0	0.0	0.0	100.0	0.0
Sarcoma, not otherwise specified (NOS)	75	71	94.4	4.2	1.4	0.0	100.0	0.0
Paraganglioma	41	37	100.0	0.0	0.0	0.0	89.2	10.8
Primitive neuroectodermal tumour (PNET)	23	18	100.0	0.0	0.0	0.0	100.0	0.0
Rhabdomyosarcoma	7	7	100.0	0.0	0.0	0.0	100.0	0.0
Schwannoma	121	107	100.0	0.0	0.0	0.0	100.0	0.0
Synovial sarcoma	12	11	100.0	0.0	0.0	0.0	100.0	0.0
Osteosarcoma	43	34	94.1	2.9	0.0	2.9	100.0	0.0
Chondrosarcoma	38	21	100.0	0.0	0.0	0.0	100.0	0.0
Total N	15306							
